# New aspects the regulation of immune response through balance Th1/Th2 cytokines

**DOI:** 10.1186/1878-5085-5-S1-A134

**Published:** 2014-02-11

**Authors:** Natalia O Tymoshok, Liudmyla M Lazarenko, Rostyslav V Bubnov, Liudmyla N Shynkarenko, Lidia P Babenko, Viktoria V Mokrozub, Yulia A Melnichenko, Mykola Ya Spivak

**Affiliations:** 1 D.K. Zabolotny Institute of Microbiology and Virology of the National Academy of Sciences of Ukraine, 154 Zabolotny str., 03143, Kyiv -143, Ukraine

## 

The ability *probiotics*, affect the relevant Toll-like receptors (TLRs), can promote effective immune response and the initiation of an effective immune defense. Gram positive bacteria affect the formation of T-and B-cell immune response by altering products primarily IFN-γ and IL-12 are required for differentiation of T helper cells into Th1 subpopulation direction. But probiotic preparations are capable of activating both (Th1 and Th2) lymphocyte subpopulations, which provides a balance of cytokine production. Immunomodulatory activity of probiotic preparations most important to identify for the goods induced opposite cytokines IL-10 or IL-12 in experiments in vitro when stimulated macrophage cells. The immune response against infectious diseases of probiotic drugs due to the ability to balance the body's immune status at the level of receptor-ligand interactions.

Probiotic preparations are the agonists TLR-2, and the presence of common protein adapter molecules (TIRAP), MyD88 for TLR-2 and TLR-4 to influence the signaling pathways of cytokine production under the influence of the ligands. Inhibition of TLR2-induced signaling, influenced by lactobacilli via adapter Mal/MyD88 can lead to partial inhibition of TLR4 signal, accompanied by decreased production of proinflammatory cytokines. Ability to influence signaling pathways of cytokine production opens new perspectives for the creation of probiotic preparations with anti-inflammatory properties.

Thus, daily oral administration of strains *L. rhamnosus V*^®^ or *L. rhamnosus* LB3 IMB B-7038 (1x10^6^ cells / mouse) for 4 days, infected *Staphyloccous aureus* 8325 mice in a dose (5x10^8^ cfu/mouse) LD50 was accompanied by a reduction in mortality of animals. The introduction of lactic acid bacteria to the animals infected with S. aureus allowed increased functional activity of phagocytic system, the normalization parameters of cellular immunity activation and production of interferon-γ and IL-12 in different periods of observation. However, decreased production of IL-4, indicating that the ability of probiotic strains to balance the immune response in the upward cytokine production by Th1-type, which guide the development of the immune response to cell type.

The introduction of lactic acid bacteria accompanied strengthening the ability of splenocytes to products IFN -α and IFN-γ in response to adequate stimulation. Strengthening biocide activity of macrophages and cytotoxicity natural killer under the influence of experimental strains leads to a significant increase of elimination S. aureus infected kidneys of animals.

## Conclusion

These data suggest that lactic acid bacteria may dynamically modulate the mechanisms of innate and adaptive immunity by maintaining the balance between Th1 and Th2 lymphocytes, which allows us to consider them as immunomodulatory drugs selective action.

